# Pneumonia

**Published:** 1883-05-20

**Authors:** 


**Pneumonia.**—Dr. Clarke, in the Chicago Medical Times, says of the treatment of pneumonia: "The entire affected section of the lung should be throughly rubbed with turpeutine and lard, *warm* (any animal oil will do, but vegetable oils, as olive oil, are not so good,) every two or three hours, according to severity of symptoms. Over this at once lay a poultice of corn meal mush, as warm and thick as can be borne, having a thin cloth or piece of mosquito bar netting between the poultice and the skin; in place of the corn meal, flaxseed may be used. Should the turpentine prove too irritating, use more lard and less turpentine. On no account suffer the skin to be injured or made so tender that the application cannot be borne until convalescence comes on. This external application is the most important part of the treatment. If the temperature is high, frequent sponge bathing, with water, at an agreeable temperature, is beneficial, and better than salicylate of soda or anything else I have ever heard of. Internally, veratrum or aconite, according to indications, with nitrate of potash and ipecac as expectorants. Milk diet, with whiskey after two or three days, in case of prostration, completes the scheme. Complications must be met by appropriate means, and I mention pleuritic pains, which may often appear, as justifying an opiate of the form best tolerated by the patient. In all, very little medicine is needed. The tendency at the present time is to give too much medicine to these cases. I have already indicated that all I have found best or necessary in any ordinary case, and must repeat that, with good, fair nursing in this climate, the cases of uncomplicated fatal pneumonia should be very rare.

**Chlorate of Potassium** in fine powder has yielded excellent results when dusted on to the surface of ulcers and ulcerating epitheliomata. The surface should be cleansed and the powder dusted thickly on, and twice a day. It relieves pain and promotes healing by changing the character of the morbid processes.—*Weekly Med. Review*, March 3d.

